# Lung cancer: principles and practice

**DOI:** 10.1054/bjoc.2001.2133

**Published:** 2001-11

**Authors:** S G Spiro

**Affiliations:** Department of Thoracic Medicine, The Middlesex Hospital, London, UK


*British Journal of Cancer* (2001) **85**(10), 1609
© 2001 Cancer Research Campaign
doi: 10.1054/ bjoc.2001.2133, available online at http://www.idealibrary.com on IDEAL

Book Review

### Lung cancer: principles and practice

**HI Pass, JB Mitchell, DH Johnson, AT Turrisi and JD Minna**

Lippincott, Williams and Wilkins 2000 pp 1117 ISBN 07817 17914 Price: £100

This is a large textbook of 1117 pages, excluding the index, going through the whole of the pathway of lung cancer from general biological concepts to the special care needs of the elderly and finally how to evaluate trial design. The book is presented in 10 parts dealing initially with the biology of lung cancer including molecular genetics post-tumour interactions, basics of therapeutics and experimental models of lung cancer. It then goes through aetiology, screening, early detection, pathology, clinical presentation including staging and prognosis, surgery, radiation therapy, chemotherapy, treatment of small cell lung cancer, palliation and special considerations, and finally statistics and trial design. The book is closely written, in small print making it a concentrated read with black and white illustrations. The chapters are of relatively uniform length so that the clinician looking for treatment advice receives as much attention as the molecular biologist looking at the various genetic characteristics of lung cancer and a chapter on gene therapy gets as much space as a chapter on chemotherapy of small cell lung cancer. Each chapter is well presented but the chapters are state of the art type reviews, virtually confined to the literature, without any very personal conclusion. The authors, in the main, are very experienced individuals in their field, but they do not step away from a concise review of the field to give an authoritative, personal or opinioned comment of the type that makes a book much more interesting to read. This is not a book that can be read from cover to cover but a book to dip in to and read individual chapters that are relevant to practice or interest. The clinician might want much more than relatively limited summaries of the literature. It is perhaps slightly irritating that minor aspects of the disease, e.g. superior vena caval obstruction and the insertion of stents, receives as much space as major areas of treatment such as chemotherapy in advanced non small cell lung cancer. The book succeeds quite well; it is let down a little by the references not being as up-to-date as they could be. The book is published in the year 2000 and there are abstracts and occasional papers referred to in 1999 which is excellent, but the relative numbers for 1996–1998 tend to be few, perhaps 3–4% of the total bibliography per chapter. The grey illustrations are hard to interpret and this applies particularly to radiographs. However, there are colour plates in the centre of the book which reproduce some of the very grey prints that appear here and there and that clearly do need colour enhancement. Why print them twice – once grey and once in colour? The references are extensive and, apart from the above caveat, satisfactory. There are slight editorial hiccups, such as in chapter 34 where 84 references appear in the text and 79 are listed.

All in all this is an authoritative and useful book. It is not an easy read and will not give you a personal view by an individual author on a particular approach to lung cancer. It is, however, a very good state of the art, reasonably up-to-date assessment of the scene as it is today. On a personal note, as a reviewer and a clinician, I would have liked to have seen much more on the common areas of controversy such as the role of chemotherapy in advanced non small lung cancer including quality of life and cost effectiveness, and more than the two short chapters on the special problems of treating the elderly patient. After all, lung cancer is increasingly a disease of the elderly.

This book will be of considerable value to respiratory physicians and oncologists dealing with lung cancer, but is probably too detailed for the general physician who comes across this disease from time to time.

**SG Spiro**
*Department of Thoracic Medicine*
*The Middlesex Hospital*
*London, UK*

